# Kupffer and stellate cell proteoglycans mediate malaria sporozoite targeting to the liver

**DOI:** 10.1186/1476-5926-2-S1-S47

**Published:** 2004-01-14

**Authors:** Gabriele Pradel, Shivani Garapaty, Ute Frevert

**Affiliations:** 1 New York University School of Medicine, Department of Medical and Molecular Parasitology, New York, New York 10010, USA

## Introduction

Malaria-infected mosquitoes inoculate *Plasmodium *sporozoites into the subcutaneous connective tissue of the mammalian host. Within minutes after entering a capillary at the bite site, thesporozoites travel to the liver where they invade their initial target cells, the hepatocytes.

Sporozoite arrest in the liver sinusoid is thought to be mediated by the specific binding of the two major *Plasmodium *sporozoite surface proteins, the circumsporozoite protein (CSP) and the thrombospondin-related adhesive protein (TRAP), to the unique heparan sulfate proteoglycans (HSPGs) of the liver [1]. Earlier work had shown that the interaction occurs between highly sulfated heparin-like oligosaccharides in heparan sulfate and two domains in CSP, a thrombospondin-like cell-adhesive region II-plus at the C-terminus and a positively charged motif upstream from the conserved region I. Similarly, the ectodomain of TRAP contains two adhesive motifs, an integrin-like A domain and a thrombospondin-like adhesive domain, that interact with host cell proteoglycans [2]. Heparan sulfate from the liver exhibits an unusually high degree of sulfation compared to heparan sulfate species from all other tissues [3]. This unique composition is most likely the reason for the remarkably selective targeting of recombinant CS protein to the liver, which occurs despite the presence of HSPGs on most other cell types. The vascular endothelium, in particular, expresses a heparan sulfate species which is clearly undersulfated.

Once arrested in the liver sinusoid, *Plasmodium *sporozoites have to cross the sinusoidal cell layer to invade hepatocytes. It was proposed that sporozoites infect hepatocytes directly by squeezing through the endothelial fenestration [4], although they exceed the maximum pore size of the sieve plates by an order of magnitude [5]. This event, however, has never been observed, nor have sporozoites been found passing through the cytoplasm of endothelial cells. Others suggested that Kupffer cells use their scavenger function to remove sporozoites from the bloodstream by phagocytosis, and that a small percentage of the parasites traverses the Kupffer cells fast enough to escape respiratory burst and lysosomal digestion and to invade hepatocytes [6].

We analyzed the proteoglycans involved in sporozoite targeting to the liver and the route of the parasites take into the liver parenchyma.

## Methods

*Plasmodium berghei *or *P. yoelii *sporozoites were isolated from the salivary glands of *Anopheles stephensi *mosquitoes and either inoculated into the portal vein of rats or incubated with primary liver cell cultures [7,8]. Liver cells were isolated by collagenase perfusion and Percoll density gradient centrifugation [9]. Sporozoite adhesion to and invasion of sinusoidal cells was distinguished by double immunofluorescence labeling and quantified [7]. The intracellular compartment harboring the parasites was analyzed by confocal and electron microscopy using markers for phagocytosis and fluid phase endocytosis [7]. A set of glycosaminoglycan lyases was used to characterize CSP and TRAP binding cell surface and extracellular matrix (ECM) proteoglycans on isolated liver cells, on cryosections of liver tissue *in situ*, and in ^35^S-sulfate labeled cell lysates and culture supernatants [8].

## Results and Discussion

We investigated the interaction between *Plasmodium *sporozoites and sinusoidal cells from rat liver. *In vivo *sporozoite invasion studies supported the notion that the parasites traverse Kupffer cells, but not endothelia [7]. Since sporozoite entry into a large organ such as the liver is an extremely elusive event, we established an *in vitro *invasion assay to characterize and quantify sporozoite adhesion to and invasion of sinusoidal cells isolated from rat liver. Using our *in vitro *model, we demonstrate that *P. berghei *and *P. yoelii *sporozoites attach to and enter Kupffer cells, but not sinusoidal endothelia. After entry into Kupffer cells, the sporozoites are enclosed in a vacuole, which does not colocalize with lysosomal markers, and remain structurally unimpaired for many hours *in vitro *suggesting that they do not elicit a respiratory burst. Inhibition of phagocytosis with gadolinium chloride, which abolishes phagocytosis of inactivated sporozoites, has no effect on the invasion of live sporozoites into Kupffer cells [7]. Thus, the sporozoites selectively recognize and actively invade Kupffer cells, avoid lysosomal fusion and phagosomal acidification, and safely pass through these stationary phagocytes of the liver.

We characterized the proteoglycan species produced by various liver cell types and found that sporozoites as well CSP and TRAP recognize distinct cell type-specific surface proteoglycans from primary Kupffer cells, hepatocytes and stellate cells, but not from sinusoidal endothelia [8]. Recombinant *P. falciparum *CSP and TRAP bind to heparan sulfate on the surface of hepatocytes and both heparan and chondroitin sulfate proteoglycans on the surface of stellate cells. On Kupffer cells, CSP recognizes predominantly chondroitin sulfate, while the TRAP binding is glycosaminoglycan-independent. *P. berghei *sporozoites attach to heparan sulfate on hepatocytes and stellate cells, whereas Kupffer cell recognition involves both chondroitin sulfate and heparan sulfate proteoglycans. CSP also interacts with secreted proteoglycans from stellate cells, the major producers of extracellular matrix in the liver. In fact, the majority of the CSP binding sites in the space of Disse are associated with these matrix proteoglycans. In agreement with our data, Gressner and Sch–fer [10] demonstrated that compared to hepatocytes, stellate cells synthesize eight times more sulfated proteoglycans and also incorporate double the amount of sulfate into heparan sulfate. In addition, stellate cells secrete more than 70% of their sulfated proteoglycans so that the vast majority of the HSPGs in the ECM derives from these cells [10]. Stellate cell-derived ECM proteoglycans are therefore prime candidates to mediate the initial arrest of malaria sporozoites in the liver sinusoid.

**Figure 1:**
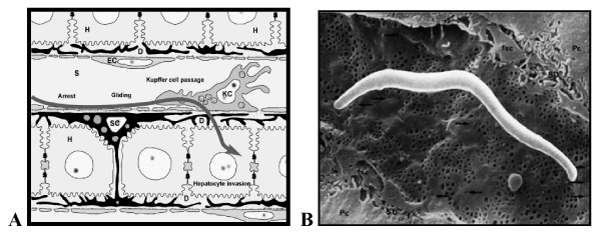
A) Model for sporozoite targeting to and entry into the liver. D = space of Disse, EC = endothelial cell, H = hepatocyte, KC = Kupffer cell, SC = stellate cell, S = sinusoid. B) Model for sporozoite arrest in the sinusoid. Due to their large size and strategic position underneath the endothelial sieve plates, stellate cell-derived ECM proteoglycans may protrude from the space of Disse across the endothelial fenestration into the sinusoidal lumen and thus provide a basis for the parasites to glide towards the next Kupffer cell.

We propose that sporozoite infection of the liver involves three steps: arrest in the sinusoid, gliding to and passage through Kupffer cells, and invasion of hepatocytes (Figure 1). Due to their large size and strategic position, stellate cell-derived ECM proteoglycans are likely to protrude from the space of Disse across the endothelial fenestration into the sinusoid thus mediating sporozoite arrest. Kupffer cell surface proteoglycans may then initiate parasite extravasation and entry into the liver parenchyma.

Kupffer cells, stationary phagocytes of the liver, play an important role in the immune surveillance of the host. Sporozoite migration through these cells should therefore generate an effective immune response against the liver stages of *Plasmodium*. However, despite an extraordinary degree of immunological challenge, immunity to natural malaria infection is acquired slowly and is rarely, if ever, complete. The finding that malaria sporozoites pass safely through Kupffer cells suggests that *Plasmodium *has developed mechanisms to interfere with the development of immunity.
